# Graphene oxide and H_2_ production from bioelectrochemical graphite oxidation

**DOI:** 10.1038/srep16242

**Published:** 2015-11-17

**Authors:** Lu Lu, Cuiping Zeng, Luda Wang, Xiaobo Yin, Song Jin, Anhuai Lu, Zhiyong Jason Ren

**Affiliations:** 1 Department of Civil, Environmental, and Architectural Engineering, University of Colorado Boulder, Boulder, CO 80309, USA; 2 Department of Mechanical Engineering, University of Colorado Boulder, Boulder, CO 80309, USA; 3 Department of Civil and Architectural Engineering, University of Wyoming, Laramie, WY 82071, USA; 4 The Key Laboratory of Orogenic Belts and Crustal Evolution, School of Earth and Space Sciences, Peking University, Beijing 100871, P.R. China

## Abstract

Graphene oxide (GO) is an emerging material for energy and environmental applications, but it has been primarily produced using chemical processes involving high energy consumption and hazardous chemicals. In this study, we reported a new bioelectrochemical method to produce GO from graphite under ambient conditions without chemical amendments, value-added organic compounds and high rate H_2_ were also produced. Compared with abiotic electrochemical electrolysis control, the microbial assisted graphite oxidation produced high rate of graphite oxide and graphene oxide (BEGO) sheets, CO_2_, and current at lower applied voltage. The resultant electrons are transferred to a biocathode, where H_2_ and organic compounds are produced by microbial reduction of protons and CO_2,_ respectively, a process known as microbial electrosynthesis (MES). *Pseudomonas* is the dominant population on the anode, while abundant anaerobic solvent-producing bacteria *Clostridium carboxidivorans* is likely responsible for electrosynthesis on the cathode. Oxygen production through water electrolysis was not detected on the anode due to the presence of facultative and aerobic bacteria as O_2_ sinkers. This new method provides a sustainable route for producing graphene materials and renewable H_2_ at low cost, and it may stimulate a new area of research in MES.

Graphene and related materials have come to the forefront of research and development due to their unique electrical, thermal, or mechanical properties and wide application potentials. For large-scale graphene production, chemical oxidation of graphite to graphite oxide or graphene oxide (GO) followed by reducing GO to graphene using chemical, thermal, or electrochemical methods has been widely studied^1^,^2^. GO not only is the important precursor for mass production of graphene-based materials^2^, it also draws extensive attention for its own potentials in many areas, including electronics and optoelectronics^3^, bio-nanotechnology^4^, renewable energy^5^, membrane research^6^ and environmental applications^7^. To date, GO is mainly synthesized by chemical oxidation based on either the Hummers, Brodie, or Staudenmaier methods^2^. All of these chemical methods use either concentrated acids, such as sulfuric acid (H_2_SO_4_) and nitric acid (HNO_3_), toxic reagents such as potassium dichromate (K_2_Cr_2_O_7_) and potassium permanganate (KMnO_4_), or even explosive potassium chlorate (KClO_3_) to oxidize graphite to GO, and the production procedure can be expensive, dangerous, and non-sustainable^1^. Electrochemical exfoliation of graphite to GO or graphene was recently performed in ionic liquids^8^,^9^,^10^, aqueous acids^11^,^12^,^13^ and inorganic salt solution^14^ under a 7−20 V voltage, and the products were reported with different levels of defect in the crystal lattice and oxygen-doping. It is possible that microorganisms can oxide dispersed graphite to graphite oxide nanosheets, but external carbon sources and oxygen were needed and the reaction rate was low^15^. Data from our preliminary studies suggests a new method for producing GO at relatively high rate through bioelectrochemical oxidization of graphite under ambient conditions.

Here we report such new and green approach to produce bioelectrochemically exfoliated graphite oxide and graphene oxide (BEGO) sheets from solid graphite rods by using a bioelectrochemical system (BES). The BEGO production is also accompanied with the production of value-added H_2_ and organic compounds. This new method eliminates the use of expensive and potentially hazardous chemicals for bulk GO production, and presents the possibility of using abundant graphite as the electron source for co-production of clean energy and chemicals. In this process, graphite rod electrode (anode) was exfoliated in a BES to produce BEGO colloidal suspension, CO_2_, and electrons. The electrons derived from the anode oxidation are transferred to a biocathode, where H_2_ and organics are produced by microbial reduction of protons and CO_2_, respectively, a process known as microbial electrosynthesis (MES)^16^,^17^ (Fig. 1A). In addition to the electricity-driven reduction of CO_2_, MES here is expanded to a more generalized process including all the microbially catalyzed synthesis of chemical compounds in an electrochemical system using direct or indirect electron transfer from the electrode to the microorganisms^17^,^18^. Bioelectrochemical system is a technology platform that uses microorganisms to catalyze electrochemical reactions for energy and chemicals production from biodegradable substrates, such as those from wastewater, soil and sediments^16^,^17^,^19^. A BES reactor traditionally uses an inert graphite anode, which serves as the electron acceptor for exoelectrogenic bacteria, also known as electricigens or anode-respiring bacteria for their anaerobic respiration. While the BES platform has been studied to produce inorganic and organic chemicals through electrolysis or electrosynthesis on the cathode^20^, the pure carbon based anode has not been reported sacrificial^16^,^21^,^22^,^23^, because it is not biologically available and poised at a low potential compared to abiotic electrochemical reactions. However, we found in this study that by coupling the biological and electrochemical mechanisms, the graphite anode could be oxidized and exfoliated into BEGO colloidal suspension at a faster rate than abiotic controls.

## Results

### Establishing an electrotrophic biocathode for electrosynthesis

The first stage of experiment aimed to establish a microbial community for electrosynthesis on the cathode. The active reactor was one time inoculated with anaerobic sludge and fed with 2 mM glucose as initial substrate, which was then operated at a fixed cathode potential of −0.6 V (vs. SHE) (Supplementary Figure S1). The system was flushed with 100% CO_2_ to reach a constant CO_2_ content of ~5.6 mmol in the headspace. Figure S1 shows that current production can be observed after 40 days and reached to 1.24 A/m^2^ at the end of batch. In contrast, only 0.01 A/m^2^ current was generated in the abiotic control reactor that was operated the same way but without inoculum. In the meantime, H_2_ was produced from the active reactor, suggesting the formation of biocatalysts reduced the potential required to overcome the real thermodynamic barrier of cathode reaction. Although the theoretical potential for H_2_ production by reduction of proton under normal condition (298K, 1 atm and pH = 7) is −0.414 V (vs. SHE), more negative potential is normally required to drive the reaction in practical due to the existence of overpotential. The slight increase of CO_2_ and the generation of CH_4_ should be due to fermentation of glucose or sludge and potentially hydrogenotrohic methanogenesis. The solution COD and pH were stable, suggesting no organic matters were produced through CO_2_ reduction at this stage. On day 57, the media were completely replaced with mineral solution without carbon source or inoculum. CO_2_ was provided again by flushing the fresh solution. Current density increased rapidly from 0.53 to 2.06 A/m^2^ from day 57 to day 77, which resulted in accumulation of 18.7 mmol H_2_ with average production rate of 9.4 mmol/L/day. Different from previous stages, significant CO_2_ reduction was observed, with the headspace CO_2_ dropped from 5.4 to 2.4 mmol. In the meantime, COD concentration in the liquid increased significantly from 96 mg/L to the 4,060 mg/L and accompanied by a pH drop from 6.78 to 4.54, indicating the formation of organic acids. In contrast, the control reactor showed no obvious current or H_2_ generation production, nor obvious CO_2_ reduction or COD accumulation. The pH kept stable in the control reactor too. Such differences suggest that an electrotrophic biocathode capable of microbial electrosynthesis is established^22^. Methanogenesis was greatly inhibited presumably due to low pH.

### Coupling graphite oxidization to BEGO with microbial electrosynthesis

After 76 days of operation, the solution was replaced again and flushed with CO_2_. As shown in Fig. 2, repeating pattern was observed, and H_2_ was the predominant product that accumulated to 110.8 mmol over 30 days. The average production rate was 36.9 mmol/L/day. No obvious CH_4_ production was observed. One key difference in this stage is the production of CO_2_ from the active reactor. CO_2_ accumulated to 35.2 mmol at an average production rate of 11.7 mmol/L/day. The solution COD continuously increased from 136 mg/L to 1350 mg/L, while the solution pH dropped from 6.76 to 5.87. No obvious gas production was observed in the abiotic control reactor, and the COD and pH kept constant. Along with CO_2_ production, gradual physical mass loss of the graphite anode and increased turbidity and color of the solution were observed in the active reactor (Fig. 3), indicating the graphite electrode was the major carbon source for CO_2_ production. Although cell decomposition and microbial metabolism also generate CO_2_, they were not dominant based on electron balance. Figure 3 shows the images of the anodes at different stages, as well as the solutions containing the exfoliated BEGO sheets. The supernatant exhibited blackish brown color. By comparison, the anode of the abiotic control reactor (cathode poised at −0.6 V vs. SHE) remained intact, and the supernatant was as clear as the fresh medium.

To double check the involvement of graphite in this bioelectrochemical reaction and understand the underlying mechanisms, we repeated the experiment by completely replacing the medium again on days 107 and 129, as well as replacing a new graphite anode on day 107. But this time we did not perform external CO_2_ flush so carbon could be balanced. Figure 2A shows the average production rates of H_2_ and CO_2_ increased further compared to the previous cycle and reached 46.3–67.8 mmol/L/day (H_2_) and 25.0–32.0 mmol/L/day (CO_2_), respectively. The ratio of accumulated volume of H_2_ to CO_2_ ranged from 1.9 to 2.1. The production of COD decreased to 545–646 mg/L at the end of each cycle, indicating that planktonic microorganisms in solution were mainly responsible for microbial electrosynthesis of organics during the enrichment period (day 57–76), where they could use indirect electron donors, such as H_2_, for electron transfer. Once they were washed away during medium replacement, less organic COD was synthesized by the biofilm attached on the electrodes. The carbon-based colloidal suspension was continuously produced in every batch cycle, which resulted in gradual loss of the graphite anode. The graphite anode lost almost 90% of its initial mass at the end of the batch (Fig. 3). Again, no change was found in the control reactor, with cathode poised at −0.6 V (vs. SHE).

In an attempt to quantify the Coulombic efficiency to measure the electron recovery in products from electrical current, we found that hydrogen production rate was highly correlated with the current density (Fig. 4b). Among the coulombs consumed, most coulombs ended in H_2_ and a small amount was used for organic synthesis (Fig. 4a). The highest total Coulombic efficiency for H_2_ and organics production using current was around 90%, indicating the energy loss due to internal resistance and cell synthesis is low. The produced H_2_ energy can offset 58–63% of total electric energy input to the system. Cyclic voltammetry (CV) profiles between the active and control reactor cathodes demonstrates that catalytic current density of active cathode was almost one order of magnitude higher than that of abiotic cathode (Fig. 4c), showing much higher electrochemical activities of the biocathode.

A maximum anode potential of +1.6 V (vs. SHE) was observed in the biotic reactor during operation, while the anode potential in the abiotic control ranged from +0.71 to +0.98 V (vs. SHE) despite a same cathode potential (−0.6 V vs. SHE) was poised on both reactors. In order to characterize the potential of abiotic electrochemical exfoliation as compared with the bioelectrochemical process, another abiotic control was operated by poising the anode (working electrode) at +1.6 V (vs. SHE) for 48 h. The current production (0.30–1.13 A/m^2^) was much lower than that in the biotic reactor (1.68–4.46 A/m^2^) despite a higher voltage (2.67 V) was applied on the abiotic reactor compared to biotic reactor (2.2 V) (Figure S3). As a result, the exfoliation rate of graphite anode was 83 mg/L/day, much lower than the biotic active reactor, which had a rate of 388 mg-BEGO /L/day.

### Characterization of BEGO

We used a suite of tools to characterize the BEGO suspension produced in the active BES reactors. The fourier transform infrared (FT-IR) spectrum of BEGO exhibited the presence of several oxygen-containing groups in the studied region but absence of any significant absorption band for graphite (Fig. 5a, S3). The groups of −OH (structural hydroxyl group, 3421–3441 cm^–1^), −COOH (carboxyl, 1730 cm^−1^), O–H (1385 cm^−1^), C–O–C (epoxy, 1221–1226 cm^−1^) and C–O (alkoxy, 1056–1060 cm^–1^) were found in both BEGO samples and CGO^24^,^25^,^26^. The peak at 1622–1635 cm^–1^ has been assigned to deformation vibration of water molecules^24^. This observation confirms the introduction of oxygen to graphite crystal and is consistent with the now widely accepted chemical composition of graphite oxide/GO^2^. The BEGO also shows typical GO hydrophilic characteristics that disperse in water but not in dimethylformamide (DMF). The UV-vis absorption spectra (Fig. 5b) of aqueous BEGO samples exhibits two characteristic features that can be used to identify graphite oxide/GO^27^: a peak at 229–232 nm (CGO is 229 nm), corresponding to π→π^*^ transitions of aromatic C–C bonds, and a shoulder at 300 nm, corresponding to n → π^*^ transitions of C=C bonds. X-ray photoelectron spectroscopy (XPS) was used to further assess the chemical composition of the BEGO (Fig. 5c). It is unreliable to use C/O ratio for evaluation of the oxidation levels of BEGO due to impossible full dehydration of a GO sample. Instead, we first deconvoluted XPS spectra of the C 1s peak into four peaks that correspond to the functional groups of C–C (carbon sp^2^, 284.5 eV), C–O (alkoxy, 285.9 eV), C=O (carbonyl, 287.4 eV) and O–C=O (carboxyl, 288.7 eV). Then, the area of each deconvoluted peak was normalized with respect to the C–C peak (Table S1). The degree of oxidation for BEGO is comparable with that indicated in microbial oxidation^15^, but both of them are less oxidized than chemical oxidized GO, suggesting that the regular structure of carbon sp^2^ domains may be easily retained during bioelectrochemical oxidation of graphite. Raman spectroscopy is a proven tool for molecular morphology characterization of carbon materials, and it reveals highly ordered graphite crystal with prominent G-band representing the planar configuration sp^2^ bonded carbon at 1583 cm^−1^ and the weak disorder band (D-band) at 1356 cm^−1^ as well as 2D-band (second order or overtone of the D-band) at 2696 cm^−1^ (Fig. 5d). The BEGO spectrum is almost identical to CGO with characteristics of broader G-band and D-band, higher intensity ratio of the D to G band (*I*_D_/*I*_G_), decrease of 2D-band and a shift of G-band to higher frequency due to amorphization of graphite^15^,^28^,^29^, indicating similar structure and composition. However, the *I*_D_/*I*_G_ intensity ratios is lower in the BEGO spectra, suggesting that BEGO has less disordered carbon atoms or larger average size of the in-plane sp^2^ domains, and therefore a higher crystalline quality than that generated by chemical oxidation^28^. The improved BEGO crystallinity is also supported by its less blue shift of G-band than CGO relative to that in graphite^29^. In BEGO, the G-band located at frequency of 1587 cm^−1^, near that in graphite (1583 cm^−1^). This indicates a less adsorption of oxygen atoms on the BEGO network.

Atomic force microscopy (AFM) images show that there are around 12 sheets (average size 300–500 nm) per 5 × 5 μm area based on the size of AFM image, and the height of sheet is 2.5–4 nm (Fig. 5e,f,g). Due to different interaction force of tapping mode for the GO and mica, the height difference is amplified at the interface. Thus, BEGO consists of 1 ∼ 3 layers of monolayer GO (~1–1.4 nm)^3^. Transition electron microscopy (TEM) images show that the BEGO displays one or few-layer structure (Fig. 5h,I, S4), which is consistent with the result of AFM. We did observe overlapped sheet structure (left side) with thickness of more than a few layers, and this is due to the lack external exfoliation means, such as sonication or mechanical stirring, commonly used in processes of chemical GO production^2^. The selected area electron diffraction (SAED) pattern (insert of Fig. 5i) of few layers BEGO shows a typical sharp crystalline ring pattern. The bright spots corresponding to the (1100) reflections are observed on the first ring came from the (1100) plane and retained the hexagonal symmetry of the (1100) diffraction pattern^30^. The observation of more than six strong hexagonal spots indicates that BEGO contains unordered crystal structure arising from oxidation^31^.

### Bacterial community involved in bioelectrochemical graphite oxidation

Bacterial community structure analysis using high throughput sequencing showed the genus *Pseudomonas* is the dominant population on the graphite anode (74% of total reads) (Fig. 6b). Other major populations include *Rhodococcus*, *Ralstonia,* and *Propionibacterium*, which accounted for 7%, 4% and 3% of the total composition, respectively. Other 90 genera making up less than 1% of total composition were found with combined total abundance of 7%. There were 5% novel genera that had not been identified yet. Some species of *Pseudomonas*, such as *P. aeruginosa*^32^*, P. alcaliphila*^33^ and *P. putida*^34^, can self-excrete redox mediators to transfer electrons to the electrode. Among *Pseudomonas* population, the majority of sequences (75.3%) were closely similar to *P. syringae* (100% similarity) (Fig. 6c), which has not been reported to carry bioelectrochemical processes of extracellular electron transfer. *Rhodococcus* is typical aromatic compound degrader by oxygenating the aromatic ring. All dominant bacterial genera are aerobic microbes except aerotolerant anaerobic *Propionibacterium*, indicating the possible oxygen production on the anode. However, the system was sealed and maintained in anaerobic condition and no molecule oxygen was detected (or below the GC detection limit) during whole process. This suggests that O_2_ may be depleted *in situ* around microbial cells. Because hydroxyl can also be resolved to generate molecule oxygen on the anode (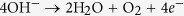) through water electrolysis, more studies are needed to understand what are the specific roles of anode microorganisms on graphite oxidation and the evolution and fate of potential O_2_.

Bacterial community on the biocathode has higher diversity than that on the anode. In addition to phylum *Proteobacteria* (anode 85%, cathode 56%), the community on the cathode was also dominated by *Fimicutes* (15%) and *Bacteroidetes* (20%), which were rarely found on the anode (Fig. 6a). *Fimicutes* was reported to be electrochemically active though the majority of such bacteria are known as *Proteobacteria*^35^. *Delftia* (11%), *Clostridium* (9%), *Alicycliphilus* (4%) and *Chryseobacterium* (12%) are dominant genera on the cathode. For *Clostridium*, all the sequences were closely similar to *C. carboxidivorans* (100% similarity), an anaerobic solvent-producing bacteria, which can grow autotrophically using H_2_/CO_2_ or CO with acetate, ethanol, butyrate and butanol as end products^36^, suggesting it may have been involved in MES process. Other dominant populations are facultative or aerobic bacteria, which likely function as O_2_ sinkers to maintain anaerobic condition in the MES.

## Discussion

The graphite oxidation in bioelectrochemical system (BES) provides a new approach for GO production, and it concurrently offers a new and abundant electron source for microbial electrosynthesis, an emerging area for value-added organic fuel and chemical production^17^,^19^,^37^,^38^. The graphite anode oxidation is hypothesized similar as the anode oxidation in molten hydroxide-based direct carbon fuel cell (MH-DCFC)^39^,^40^ (Equation 1).


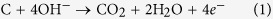


However, operation of MH-DCFC requires high temperature (500–650 °C), while the processes in this study occurred at room temperature. The CO_2_ should be generated from graphite oxidation, because no other carbon source was available in the medium. Similarly, the electrical current was also primarily derived from graphite oxidation, because no apparent O_2_ was detected in the system, which indicates limited possibilities of electron generation by water electrolysis. Reduction of O_2_ by electrons at the cathode is not believed to be the main O_2_ sink due to extreme high Coulombic efficiency (90%) for H_2_ and organics production. Hydrolysis driven by bioelectrochemical reactions provided hydroxyl and proton for graphite anode oxidation and H_2_ generation at the cathode, respectively (Eq. 2).


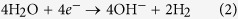


This hypothesis is supported by the observed 2:1 production ratio between H_2_ and CO_2_ (Fig. 2, Eqs. 1 and 2), and the balanced consumption of both protons and hydroxyl ions described in Equations 1–2 is supported by the observation of stable pH during the whole experiment. The standard Gibbs free energy change of whole reaction 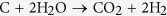 is positive, indicating it is a non-spontaneous process. A theoretical minimum voltage of 0.196 V has to be applied in order for the reaction to proceed under standard biological condition (T = 298.15 K, P = 1 bar, pH = 7) here based on thermodynamic calculation (Supplementary Information). However, much higher external energy input (>2.0 V) was needed in practice to overcome the overpotential loss at both electrodes.

In the BES reactors, microorganisms served as the biocatalysts to reduce the over-potential of reaction on the electrode and significantly enhanced current production compared to the abiotic control reactors. Microbial community structure shows that the majority of bacteria on the anode were heterotrophs. Thus, the graphite carbon was not used as carbon source, but rather the microbes utilized the organics produced from the MES. Both H_2_ and organics produced during MES could provide the energy source for the growth of anode microorganisms. While the specific contribution of microbial activities on GO exfoliation needs further investigation, it was clear that with bacteria the exfoliation rate was much higher. While a cathode potential was poised (−0.6 V vs. SHE), the anode potential of the reactor increased to a maximum of  + 1.6 V as compared with the abiotic control (0.71–0.98 V). This change should be associated with the biofilm catalysts formation on the cathode that reduced activation energy barrier, because the current in active reactor was four times that in the abiotic control. In addition, solution chemistry such as pH and ionic composition were changed due to microbial metabolisms, which also impacts GO exfoliation. Therefore, we hypothesize the mechanisms of bioelectrochemical exfoliation of graphite for BEGO production include: (i) Hydroxyl may act as a nucleophile to attack the edge and grain boundaries of graphite^14^ to open the sites up. (ii) Intermediates of microbial metabolisms such as electrosynthesis organics, a small amount of salt ions, and water may intercalate into the graphitic layers and cause expansion of graphite interlayers, and (iii) CO_2_ produced during process can also exert large force into the graphite layers to exfoliate them from one another.

The electrotrophic biofilm established on the cathode or planktonic cells utilized CO_2_ and electrons from the cathode or H_2_ for microbial electrosynthesis, which resulted in the accumulation of organic compounds as reflected by COD. The H_2_ production rate (46.3–67.8 mmol/L/day) was more than twice that of the previous BES studies (17.5–25.3 mmol/L/day) using water or reduced ferricyanide as electron sources at lower cathode potential (–0.8 to –0.7 V vs. SHE)^41^,^42^ and we attribute this to the higher current density (4.5 A/m^2^) by graphite oxidation compared to 1.1–2.4 A/m^2^ in previous systems. Hydrogenotrophic methanogenesis was effectively inhibited despite the presence of large amount of H_2_ and CO_2_, which was likely due to the low pH (4.54) during acclimation stage. It is widely accepted that most methanogens prefer a neutral environment for metabolism^43^.

The unique properties of graphene materials have been well recognized for their application potentials, and researchers around the world are diligently developing new methods to produce lower cost, high quality, and mass amount of graphene materials. The European Commission recently committed an unprecedented €1 billion Euros investment to graphene research (http://graphene-flagship.eu/), calling it the “the largest research excellence awards in history”, and it is forecasted that the graphene material markets will grow from $20 million in 2014 to more than $390 million in 2024^44^. The preliminary economic analysis shows the cost of GO production through bioelectrochemical route can be orders of magnitudes lower than current methods (Supplementary Information). While this study reports the first findings of such a method, more studies are needed to understand the mechanisms, characterize microbial and electrochemical functions, test different materials, and optimize system configuration and performance. Some specific tasks may include more precise carbon and electron balance studies, system characterizations under different potentials, microbial communities, effects of GO on microbial growth and metabolisms, and materials purification and system optimization.

## Methods

### Reactor construction and operation

**Figure 1:**
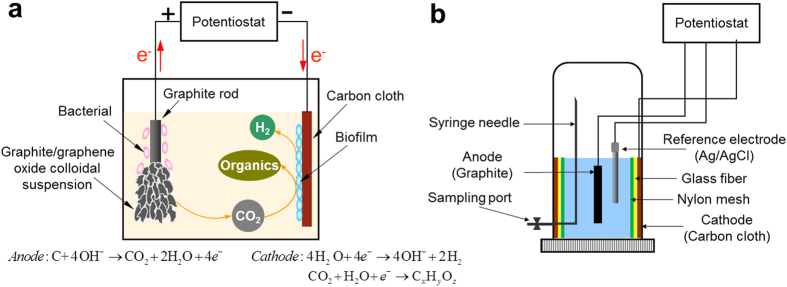
Schematic illustration of 3-electrode bioelectrochemical system (BES). (**a**) The hypothesized reaction mechanisms in BES, including microbial catalyzed GO production on the anode and microbial electrosynthesis on the cathode. (**b**) The configuration of water sealed anaerobic BES reactors.

**Figure 2:**
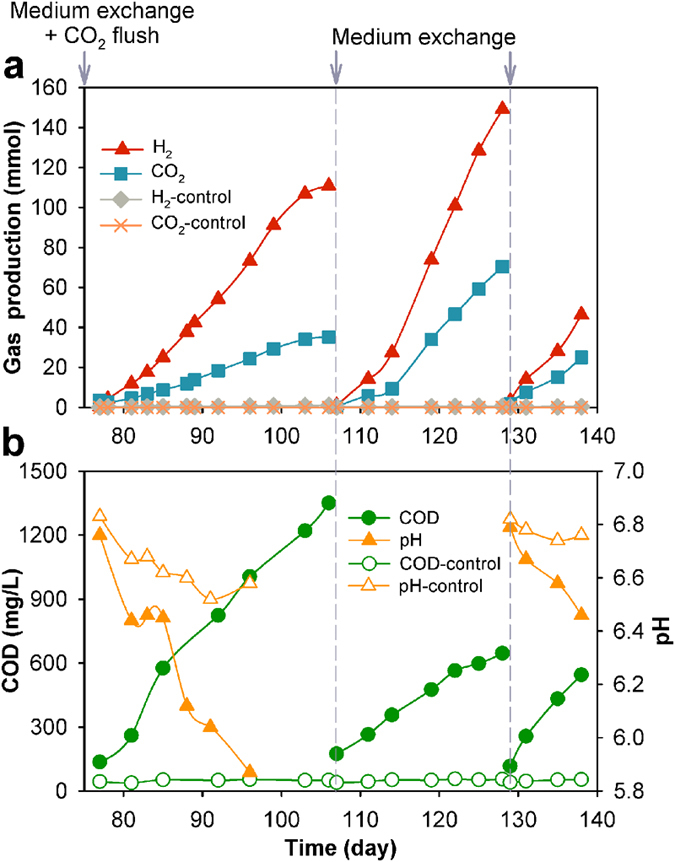
Products generation during BEGO production and microbial electrosynthesis. (**a**) Gas (H_2_ and CO_2_) production in the BES. (**b**) Changes of COD and pH of the solution. Cathodes were poised at −0.6 V (vs. SHE). Uninoculated abiotic reactor with the cathode poised at −0.6 V vs. SHE was used as control. Data shown from day 76 to day 138, representing repeatable cycles of system performance after microbial acclimation and enrichment. Results of acclimation period from day 1 to day 76 can be found in Supplementary Information (Figure S1).

**Figure 3:**
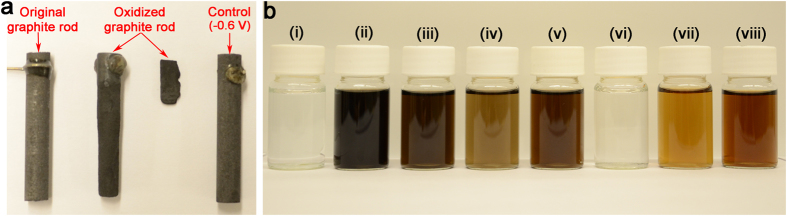
Digital images of (**a**) graphite rods before and after bioelectrochemical exfoliation, and (**b**) exfoliation production mixture dispersed in phosphate buffer medium for three weeks. (**i**) fresh phosphate medium; (**ii**) original sample from active reactor; (**iii**,**iv**) 2- and 4- fold dilution of sample ii, respectively; (**v**,**vi**) samples from abiotic control reactors with anode poised at +1.6 V (vs. SHE) and cathode poised at −0.6 V, respectively; (**vii**,**viii**) purchased chemical graphene oxide (CGO) dispersion in H_2_O (Sigma-Aldrich) with concentrations of 0.2 and 0.4 mg/mL, respectively.

**Figure 4:**
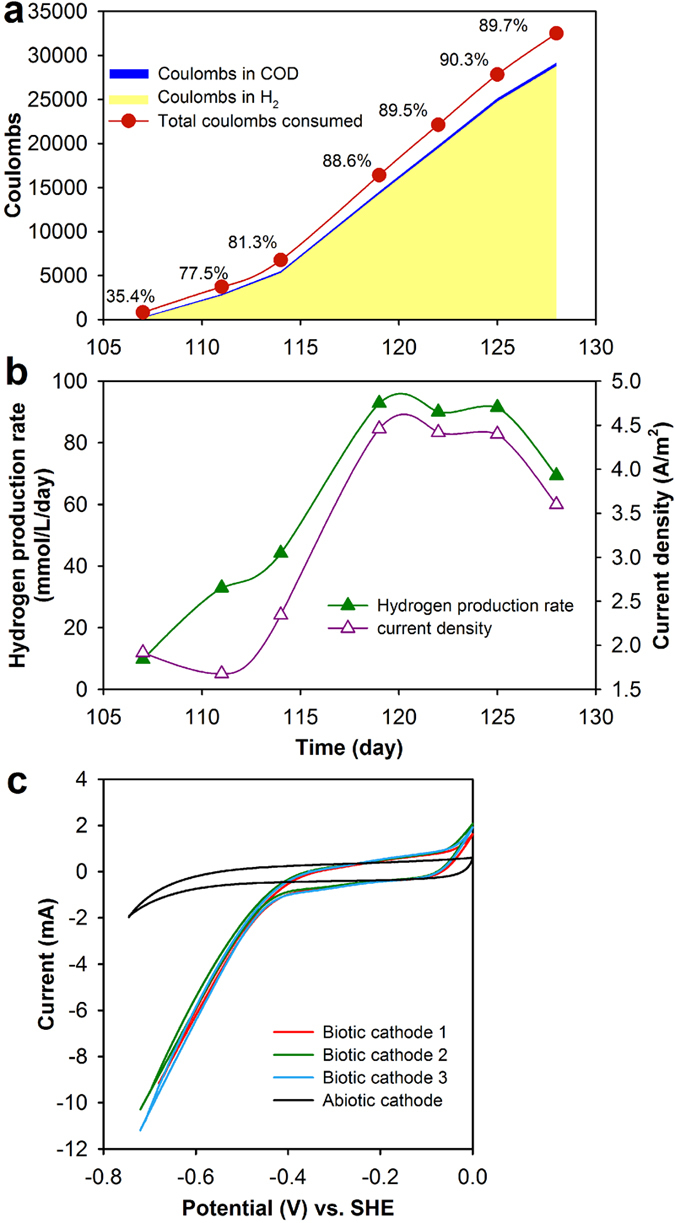
System electron balance and bacterial electrochemical activities. (**a**) Distribution of coulombs in products (H_2_ and organics) compared to total coulombs consumed. (**b**) The positive correlation between H_2_ production rate and current density (cathode poised at −0.6 V vs. SHE). (**c**) Cyclic voltammograms (1 mV/s) for the biotic cathode (active reactor, three reproducible scans) and abiotic cathode (control reactor).

**Figure 5:**
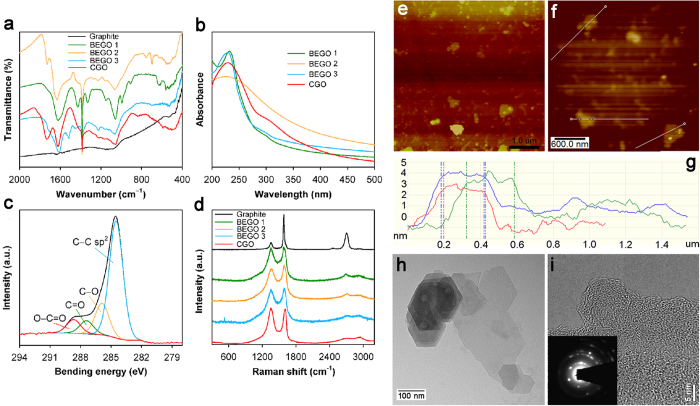
Characterization of the produced BEGO. (**a**) FT-IR spectra of BEGO samples, graphite powder obtained from raw electrode and purchased chemical graphene oxide (CGO). (**b**) UV-vis spectra of BEGO and CGO in aqueous solutions. (**c**) XPS of C 1s spectra of BEGO. (**d**) Raman spectra of BEGO, graphite and CGO. (**e**) AFM image, (**f**) AFM zoomed image for the middle region, and (**g**) AFM cross-section height profile. (**h**) TEM and (**i**) HRTEM images. The inset of (**i**) is the selected area electron diffraction (SAED) pattern.

**Figure 6:**
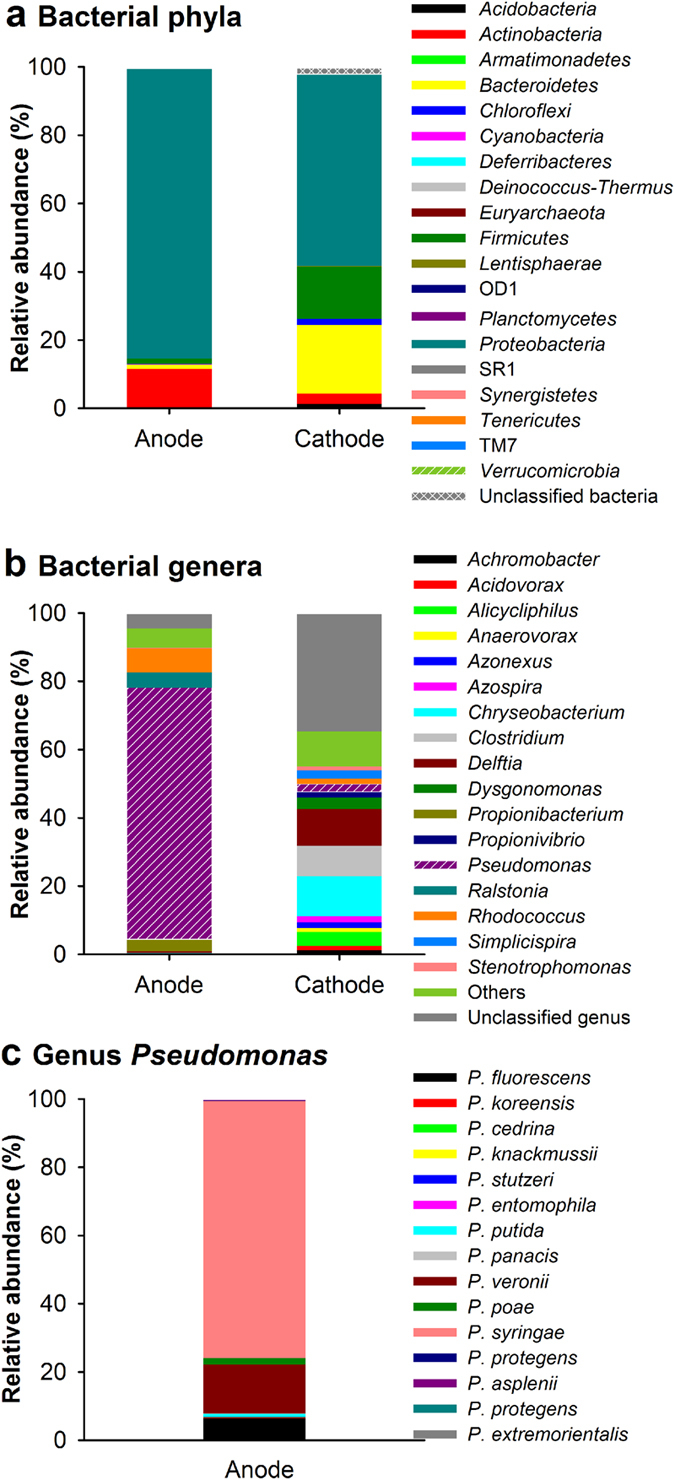
Bacterial community composition on the anode and cathode. Dominant bacterial (**a**) phyla and (**b**) genera. (**c**) Distribution of specific species (identity >97%) within genus of *Pseudomonas* on the anode. Genera making up less than 1% of total composition were classified as “others”.

Cylindrical glass bottles are used as three-electrode reactor systems. Each reactor has an effective volume of 220 mL (Fig. 1B). Graphite rod (D 0.6 cm × L 5 cm, Graphite Sales, OH) was used as the anode, and carbon cloth (5 cm x 10 cm, Fuel Cell Earth, MA) served as the cathode. A layer of nonconductive permeable glass fiber and a layer of nylon mesh were placed onto cathode as separator and support^45^. An Ag/AgCl reference electrode (RE-5B, BASi) was placed in between the anode and the cathode (0.198 V vs SHE). The electrodes were connected using titanium wires. A potentiostat (CHI1000B, CH Instruments Ins.) was used to poise the cathode (working electrode) at −0.6 V (vs. SHE), where anode was the counter electrode. All potentials are reported versus SHE.

The active reactors were inoculated with anaerobic sludge collected from an anaerobic digester, and the initial medium include (per liter): 0.36 g of glucose, 11.55 g of Na_2_HPO_4_∙12H_2_O, 2.77 g of NaH_2_PO_4_∙2H_2_O, 0.31 g of NH_4_Cl, 0.13 g of KCl, and trace materials and vitamins^46^ flushed with 100% CO_2_ to reach a constant CO_2_ content of  ~ 5.6 mmol in the headspace. Carbon dioxide was used as carbon source for microbial electrosythesis of organics. When current production was observed, the liquid was replaced with the same medium but without glucose and sludge inoculum. The operation was conducted in anaerobic condition, and after each transfer, the liquid and headspace were flushed with 100% CO_2_. The enrichment stage was considered complete when high current, H_2_ and organic production were observed. After that at least three batch cycles were operated. To quantify carbon flow, no more CO_2_ flushing was conducted during media change after the first cycle. The exfoliated anode would be replaced if necessary during this stage. Three reactors were prepared as experiment replicates but were started at different time. The three reactors showed similar operation profiles and one reprehensive time course reactor profile is reported. The BEGO1, BEGO2 and BEGO3 are samples taken from three replicated reactors, respectively. All reactors were operated at room temperature of 25 ± 2 °C. Two abiotic control reactors were operated. One followed the same protocol as the active reactor, with the cathode poised at −0.6 V (vs. SHE). Another control was setup by poising the anode at +1.6 V (vs. SHE), which was the maximum anode potential observed in biotic BES reactor.

### Chemical analysis and calculation

Gas production was continuously collected using a gas bag (1L, Cali-5-Bond, Calibrated Instruments Inc.) connected to the headspace through a syringe needle. H_2_ and CH_4_ were measured by a gas chromatograph (Model 8610C, SRI Instruments) equipped with a thermal conductivity detector with nitrogen as the carrier gas. CO_2_ and O_2_ were analyzed using helium as the carrier gas^47^. Liquid samples were filtered through 0.22 μm membrane and then centrifuged at 13,000 rpm for 1 h to remove impurities such as carbon nanoparticles and bacteria. For organic product measurement, soluble chemical oxygen demand (COD) was analyzed using a standard method (TNTplus COD Reagent; HACH Company). Cyclic voltammetry (CV) was conducted at a scan rate of 1 mV/s between −0.8 to 0 V (vs. SHE) using a separate potentiostat (PC4/300, Gamry Instruments, NJ). The scan was carried out several times and the stable CV curves were used for analysis. The pH was measured using a pH meter (HQ440d, HACH company).

Total coulombs consumed (*C*_T_) based on current measurements were calculated by integrating the current over time. Coulombs found in the product (*C*_P_) was calculated using equation *C*_p_ =*n*·*b*·F, where *n* is the moles of product, *b* is the number of electrons in the product (*b* = 2 and 4 for per mole of hydrogen and COD, respectively), and F is Faraday’s constant (96,485 C/mol). Coulombic efficiencies were calculated by dividing *C*_P_ by *C*_T_. Gas production rate (mmol/L/d) and current density (A/m^2^) was calculated on the basis of solution volume (100 mL) and cathode projected area (50 cm^2^), respectively. The amount of energy (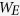) added to the circuit by the potentiostat over a period of time (*t*) was calculated by equation 
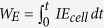
, where *I* and *E*_*cell*_ are current in the circuit and the voltage over the BES, respectively. The electricity energy consumption was normalized to kilowatt-hours (kWh) via 3600 kilojoules(kJ) per kWh.

### Characterization of BEGO

The exfoliation mixture was first centrifuged at 2500 rpm to remove large graphite particles and possible planktonic microorganisms, and then the supernatant was filtered through a 0.1 μm Durapore PVDF filter (Millipore Co.,). The remaining solid materials on the filter were washed with the following sequence: 18 MΩ water, 80% ethanol, 18 MΩ water, 1 N HCl, and 18 MΩ water again. The resulting materials called BEGO were re-suspended in 18 MΩ water for characterization. UV-vis absorption spectra were recorded using a Thermo GENESYS 10S UV-vis spectrophotometer. Fourier transform infrared spectroscopy (FT-IR) was performed by using a Thermo-Nicolet FT-IR Avatar 370 spectrometer. XPS analysis was carried out by a Kratos Axis His spectrometer. Raman spectra were obtained by Raman Microscope with wavelength of 532 nm and a 100 × objective. Atomic force microscopy (AFM) images were generated on a VEECO Dimension 3100. Transition electron microscopy (TEM) and electron diffraction images were obtained using a FEI Tecnai G20 electron microscope. Samples for AFM and TEM imaging were prepared by drop-casting the dispersion onto freshly cleaved mica substrates and lacey carbon TEM grid, respectively, which were then air dried under ambient lab conditions.

### Bacterial community composition analysis

Anode biofilm samples were scratched using a sterilized blade, and small amount of carbon cloth cathode was cut using a sterile scissor. Both of them were used for total genomic DNA extraction using a PowerSoil DNA Isolation Kit (MoBio Laboratories, Inc., CA), and bacterial 16S rRNA gene targeted at the hypervariable V1-V3 region was amplified by PCR using a 10-nucleotide barcoded forward primer 8F (5′-AGAGTTTGATCCTGGCTCAG-3′) and the reverse primer 533R (5′-TTACCGCGGCTGCTGGCAC-3′) for high-throughput 454 GS-FLX pyrosequencing. The pyrosequencing and bioinformatics analysis were carried out according to our previous description^46^,^48^. Pyrosequencing produced 6816 and 6855 qualified sequences with an average length of 450 bp for anode and cathode community, respectively. Raw sequencing data were deposited to the NCBI Sequence Read Archive (SRA) with accession No. SRP040685.

## Additional Information

**How to cite this article**: Lu, L. *et al.* Graphene oxide and H_2_ production from bioelectrochemical graphite oxidation. *Sci. Rep.* **5**, 16242; doi: 10.1038/srep16242 (2015).

## Supplementary Material

Supplementary Information
