# Supplementary material for: Graphene oxide and H2 production from bioelectrochemical graphite oxidation
Source: Sci Rep. 2015 Nov 17;5:16242. doi: 10.1038/srep16242 (PMC4647224; doi:10.1038/srep16242)
Supplement: Supplementary Information [file srep16242-s1.doc]

Page: 6; Figure: 4; Table: 1

Supplementary Information for

**Graphene oxide and H_2_ production from bioelectrochemical** **graphite oxidation**

Lu Lu^1^, Cuiping Zeng^1,4^, Luda Wang^2^, Xiaobo Yin^2^, Song Jin^3^, Anhuai Lu^4^, Zhiyong Jason Ren^1^*

^1^Department of Civil, Environmental, and Architectural Engineering, University of Colorado Boulder, Boulder, CO 80309, USA

^2^Department of Mechanical Engineering, University of Colorado Boulder, Boulder, CO 80309, USA

^3^Department of Civil and Architectural Engineering, University of Wyoming, Laramie, WY 82071, USA

^4^The Key Laboratory of Orogenic Belts and Crustal Evolution, School of Earth and Space Sciences, Peking University, Beijing 100871, PR China

*Corresponding author Ren E-mail: jason.ren@colorado.edu; phone: (303) 492-4137; fax: (303) 492-7217


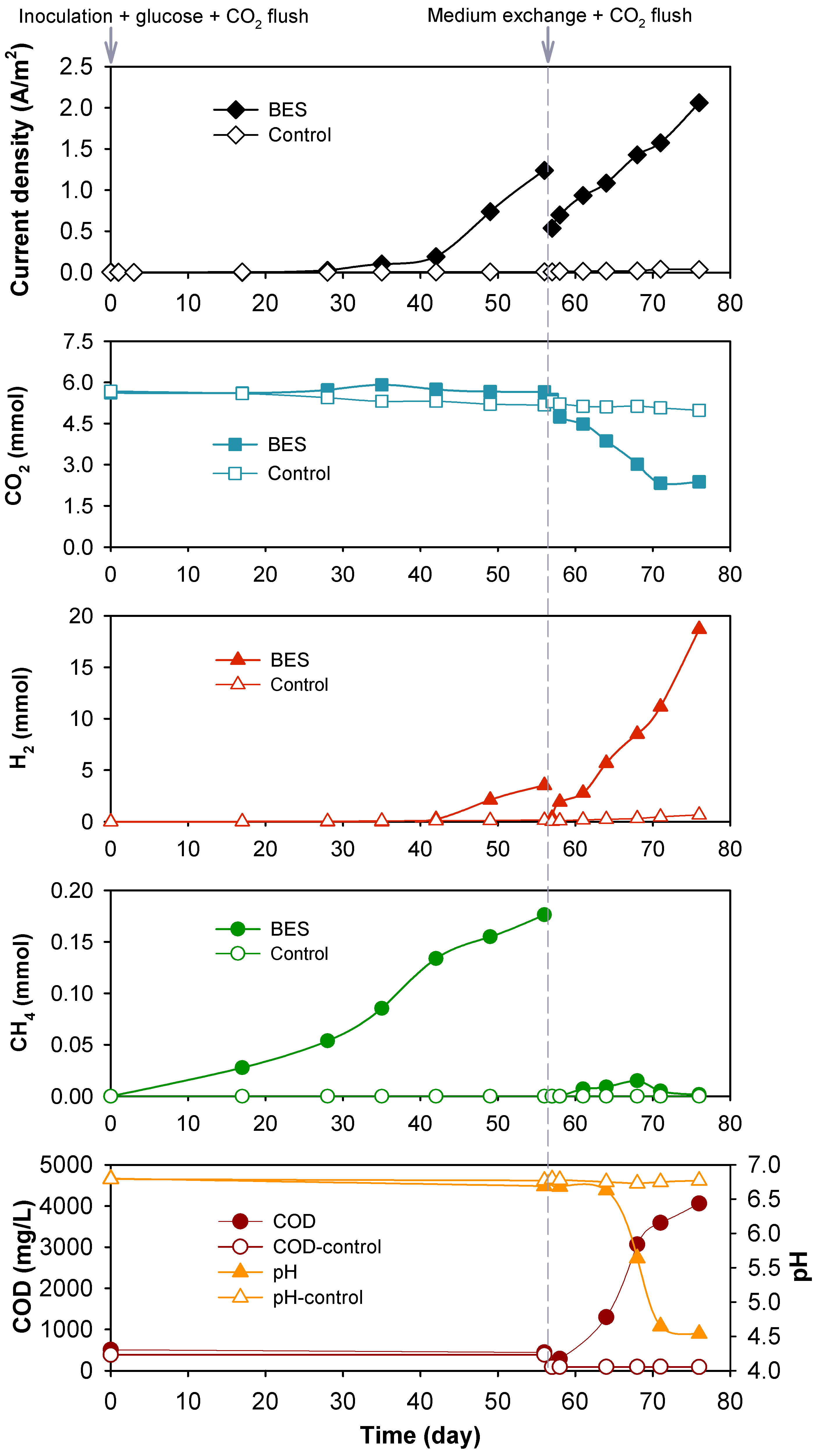


**Figure S1** Changes in current density, gas composition, COD and pH in BESs with cathodes poised at –0.6 V (vs. SHE) during enrichment of electrodes from 0 to 76 days. Uninoculated abiotic reactor with the cathode poised at –0.6 V vs. SHE was used as control.


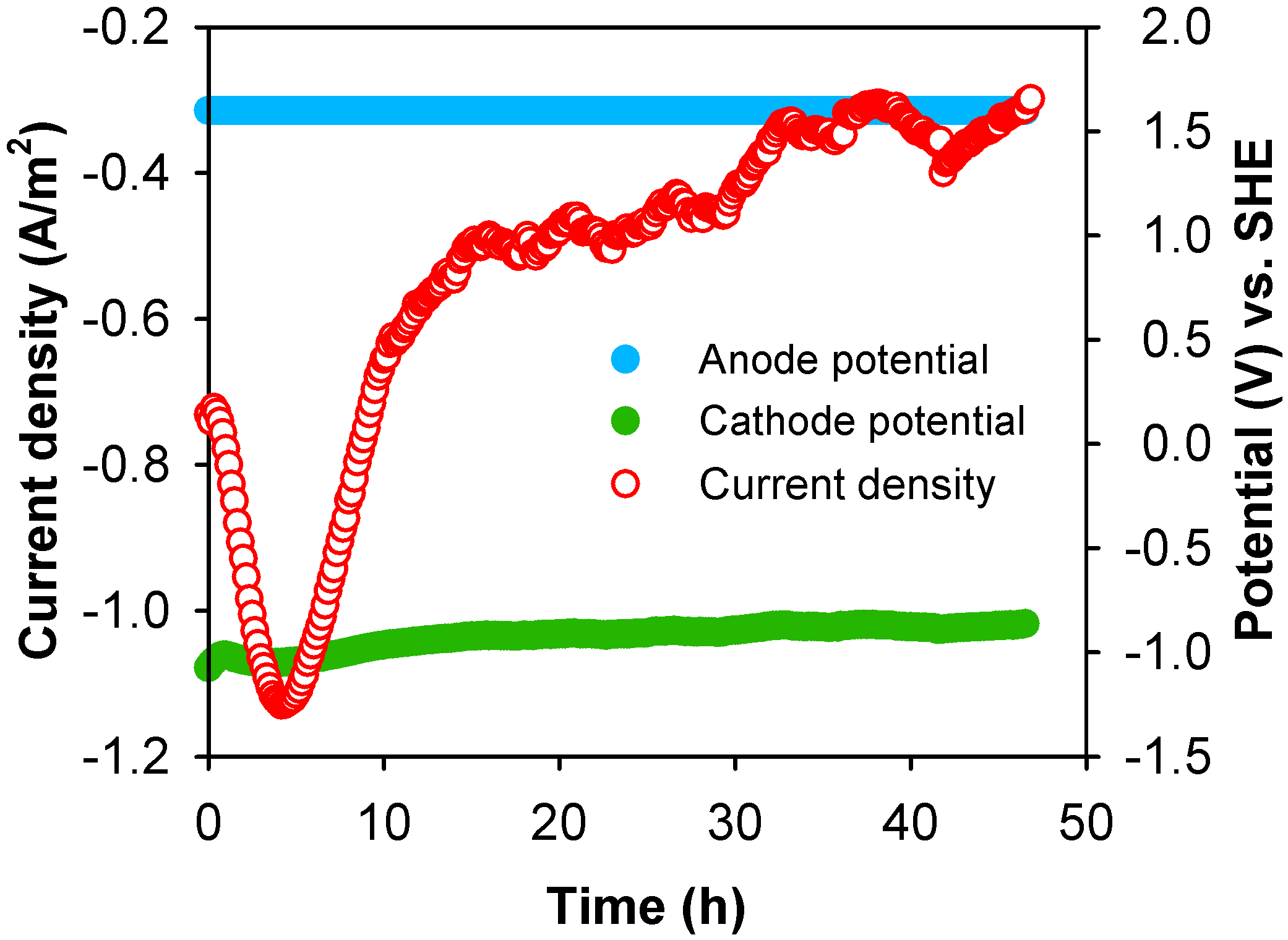


**Figure S2** Current density and electrode potential of abiotic control reactor with anode poised at +1.6 V vs. SHE.


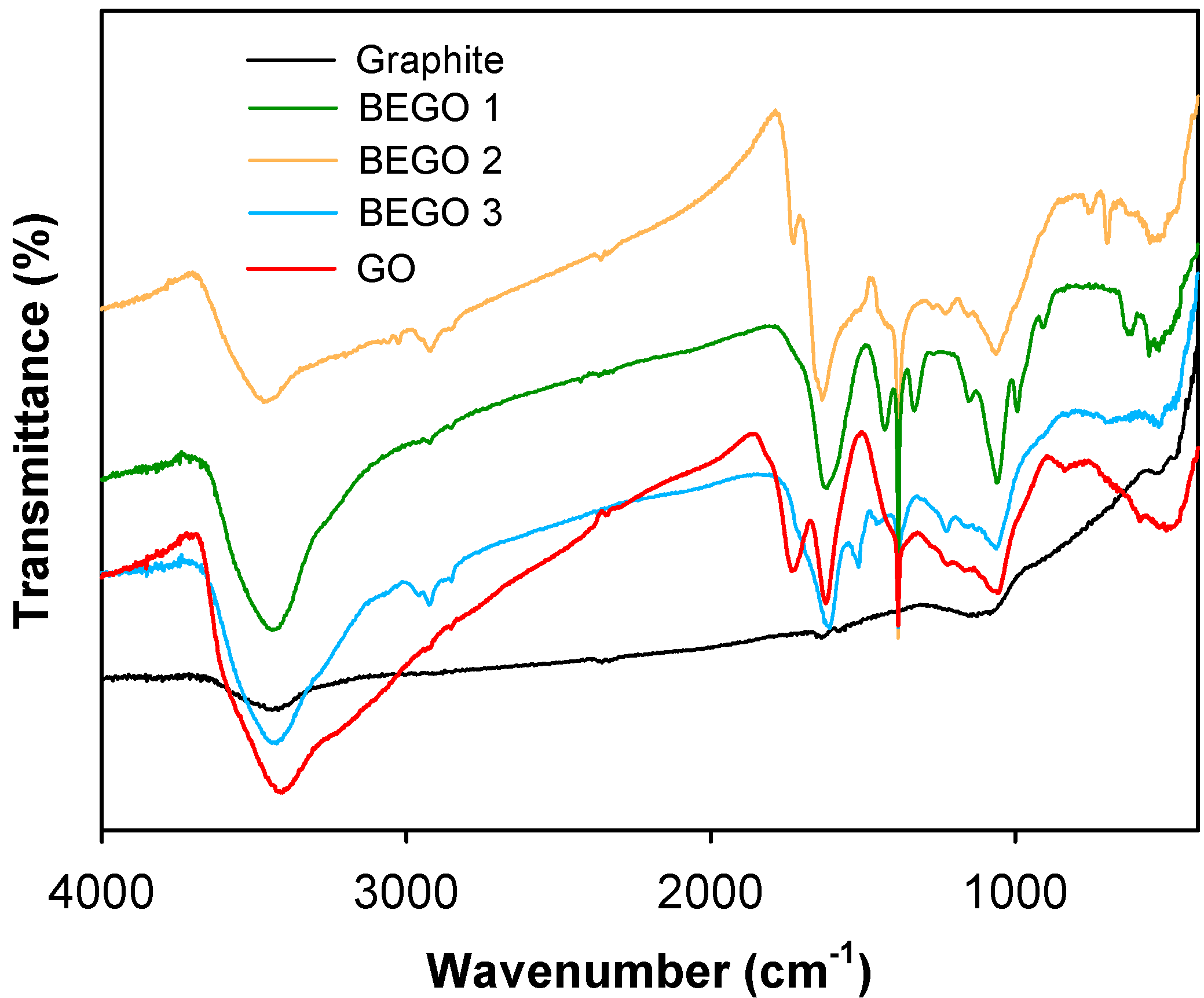


**Figure S3** FT-IR spectra of BEGO samples, graphite powder obtained from raw electrode and purchased chemical graphene oxide (CGO).


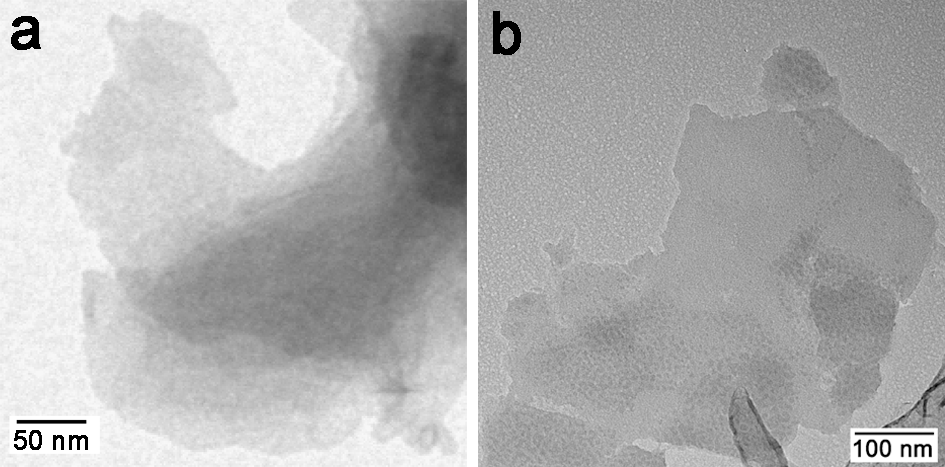


**Figure S4** TEM images of BEGO.

**Thermodynamic Properties of Hypothetical Bioelectrochemical reactions**

In this study all reactions were carried out under standard biological conditions ( T = 298.15 K, P = 1 bar, pH = 7). According to the IUPAC convention, standard potentials are reported as a reduction potential, so the reaction at the anode is written as consuming electrons:

$$\mathrm{CO}_{2}+2\text{H}_{2}\text{O}+4e^{-}\to\mathrm{C}+4\mathrm{OH}^{-}$$

Changes in standard Gibbs free energy

$$\begin{matrix} \Delta G_{r}^{0}=4\Delta_{f}G^{0}(\mathrm{OH}^{-})-[\Delta_{f}G^{0}(\mathrm{CO}_{2})+2\Delta_{f}G^{0}(\mathrm{H}_{2}\mathrm{O})] \\ =4\times(-157.20)-[(-394.37)+2\times(-237.14)]= 239.85 \mathrm{kJ}/\mathrm{mol} \end{matrix}$$

Standard anode potential

$$E_{An}^{0}=-\frac{\Delta G_{r}^{0}}{nF}=-\frac{239850}{4\times96485}=-\text{0.621 V}$$

So, the theoretical anode potential

$$\begin{matrix} E_{An}=E_{An}^{0}-\frac{RT}{nF}\ln\left(\frac{[\mathrm{OH}^{-}]^{4}}{p[\mathrm{CO}_{2}]}\right) \\ =-0.621-\frac{8.31447\times298.15}{4\times96485}\ln\left(\frac{\left(10^{-7}\right)^{4}}{0.3333}\right)=-0.214\mathrm{V} \end{matrix}$$

The reaction at the anode is written as:

$$4\mathrm{H}_{2}\mathrm{O}+4e^{-}\to4\mathrm{OH}^{-}+2\mathrm{H}_{2}$$

Changes in standard Gibbs free energy

$$\begin{matrix} \Delta G_{r}^{0}=4\Delta_{f}G^{0}(\mathrm{OH}^{-})-4\Delta_{f}G^{0}(\mathrm{H}_{2}\mathrm{O}) \\ =4\times(-157.20)-4\times(-237.14)= 319.76\mathrm{kJ}/\mathrm{mol} \end{matrix}$$

Standard anode potential

$$E_{Cat}^{0}=-\frac{\Delta G_{r}^{0}}{nF}=-\frac{319760}{4\times96485}=-\text{0.829 V}$$

So, the theoretical anode potential

$$\begin{matrix} E_{Cat}=E_{Cat}^{0}-\frac{RT}{nF}\ln\left([\mathrm{OH}^{-}]^{4}p[\mathrm{H}_{2}]^{2}\right) \\ =-0.829-\frac{8.31447\times298.15}{4\times96485}\ln\left(\left(10^{-7}\right)^{4}0.6666^{2}\right)=-0.410\mathrm{V} \end{matrix}$$

The equilibrium voltage can be calculated as:

$$E_{\mathrm{eq}}=E_{\mathrm{cat}}-E_{\mathrm{an}}=\left(-0.410\right)-\left(-0.214\right)=-0.196\mathrm{V}$$

The negative sign indicated that a voltage need to apply to overcome this thermodynamic limit.

**Table S1**. XPS data of C 1s of BEGO deconvoluted into individual peaks: bending energy and relative peak area percentages with respect to C–C peak (in parentheses). These data were compared to that reported in literature using microbial or chemical methods for synthesis of GO.

| Sample | Synthesis approach | C–C | C–O | C=O | C–OH | C–O–C | O–C=O | Literature |
| --- | --- | --- | --- | --- | --- | --- | --- | --- |
| BEGO | Bioelectrochemical oxidation | 284.5 (100) | 285.9 (27) | 287.4 (11) |  |  | 288.7  (12) | This study |
| MGO | Microbial oxidation | 284.8 (100) |  |  | 286.1 (28) | (0) | 288.6  (16) | [^1^](#_ENREF_1) |
| CGO1 | Chemical oxidation  (24 h of oxidation) | 285.1 (100) |  |  | 286.9 (76) | 287.6 (36) | 289.0 (217) | [^2^](#_ENREF_2) |
| CGO2 | Chemical oxidation  (48 h of oxidation) | 284.9 (100) |  |  | 286.5 (12) | 287.8 (46) | 289.6  (31) | [^2^](#_ENREF_2) |

**BEGO Production Rate, Yield and Economics**

We calculated BEGO production rate by normalizing mass to the volume of medium solution (100 mL) and reaction time. For the period of day 107～138, the average BEGO production rate is 388 mg/L/day with a yield of 42% relative to the total weight of the starting graphite weight. This value is almost 8 times to GO nanosheet production obtained through microbial oxidation of graphite particle suspension[^1^](#_ENREF_1).

There is very limited information in literature discussing the cost for producing graphene materials using different methods. The market price of graphene oxide varies from a few thousand dollars per kg GO to tens of thousands of dollars. Although a precise assessment of the cost for large scale BEGO production is not available at current stage, based on the lab scale study, the electric energy consumption is 23 kWh per kilogram BEGO produced. However, 29～32% of such energy can currently be offset by the produced H_2_ energy, assuming 50% efficiency in hydrogen fuel cells. Therefore, the energy consumption can decrease to 15.64 kWh per kilogram BEGO produced, which is orders of magnitudes lower than electrochemical processes[^3-9^](#_ENREF_3). This means for producing 1 kg of BEGO, the energy cost is only $1.142 based on current industrial electricity price of $0.073/kWh (June 2014)[^10^](#_ENREF_10). Assuming the price of large flake graphite (94～97% C) is $1300/t (http://northerngraphite.com/graphite-labs/graphite-price/), the graphite cost would be $3.1/kg-BEGO (42% yield from graphite). Though there is no reported data on capital cost of similar systems, our preliminary calculation based on the cost of advanced alkaline electrolyzer ($0.6/kg of H_2_ produced)[^11^](#_ENREF_11) and the H_2_ yield (0.315 kg-H_2_/kg-BEGO) estimated that a capital cost is $0.189/kg-BEGO. Assuming maintenance charge is 3% of capital cost[^11^](#_ENREF_11), or $0.006/kg-BEGO, the total gross cost of such a system is then $4.437/kg-BEGO, orders of magnitudes lower than current prices. Moreover, the BEGO is produced under ambient condition without using any chemicals, which significantly avoided the cost of hazardous waste disposal and reduces the overall environmental impacts of the process. Further economic and environmental impact analyses need to be conducted for better understanding and quantification.

**References**

1. Zhu, C., Hao, Q., Huang, Y., Yang, J.&Sun, D. Microbial oxidation of dispersed graphite by nitrifying bacteria 2011.2. *Nanoscale* **5**, 8982−8985 (2013).

2. Jeong, H.-K., Lee, Y. P., Lahaye, R. J., Park, M.-H., An, K. H., Kim, I. J., Yang, C.-W., Park, C. Y., Ruoff, R. S.&Lee, Y. H. Evidence of graphitic AB stacking order of graphite oxides. *J. Am. Chem. Soc.* **130**, 1362−1366 (2008).

3. Liu, N., Luo, F., Wu, H., Liu, Y., Zhang, C.&Chen, J. One-step ionic-liquid-assisted electrochemical synthesis of ionic-liquid-functionalized graphene sheets directly from graphite. *Adv. Funct. Mater.* **18**, 1518−1525 (2008).

4. Lu, J., Yang, J.-x., Wang, J., Lim, A., Wang, S.&Loh, K. P. One-pot synthesis of fluorescent carbon nanoribbons, nanoparticles, and graphene by the exfoliation of graphite in ionic liquids. *ACS nano* **3**, 2367−2375 (2009).

5. Singh, V. V., Gupta, G., Batra, A., Nigam, A. K., Boopathi, M., Gutch, P. K., Tripathi, B. K., Srivastava, A., Samuel, M.&Agarwal, G. S. Greener electrochemical synthesis of high quality graphene nanosheets directly from pencil and its SPR sensing application. *Adv. Funct. Mater.* **22**, 2352−2362 (2012).

6. Su, C.-Y., Lu, A.-Y., Xu, Y., Chen, F.-R., Khlobystov, A. N.&Li, L.-J. High-quality thin graphene films from fast electrochemical exfoliation. *ACS nano* **5**, 2332−2339 (2011).

7. Parvez, K., Li, R., Puniredd, S. R., Hernandez, Y., Hinkel, F., Wang, S., Feng, X.&Müllen, K. Electrochemically exfoliated graphene as solution-processable, highly conductive electrodes for organic electronics. *ACS nano* **7**, 3598−3606 (2013).

8. Parvez, K., Wu, Z.-S., Li, R., Liu, X., Graf, R., Feng, X.&Müllen, K. Exfoliation of Graphite into Graphene in Aqueous Solutions of Inorganic Salts. *J. Am. Chem. Soc.* **136**, 6083−6091 (2014).

9. Liu, J., Yang, H., Zhen, S. G., Poh, C. K., Chaurasia, A., Luo, J., Wu, X., Yeow, E. K. L., Sahoo, N. G., Lin, J.&Shen, Z. A green approach to the synthesis of high-quality graphene oxide flakes via electrochemical exfoliation of pencil core. *RSC Adv.* **3**, 11745−11750 (2013).

10. Electric Power Monthly with Data for June 2014. U.S. Energy Information Administration (2014).

11. Rau, G. H., Carroll, S. A., Bourcier, W. L., Singleton, M. J., Smith, M. M.&Aines, R. D. Direct electrolytic dissolution of silicate minerals for air CO_2_ mitigation and carbon-negative H_2_ production. *Proc. Natl. Acad. Sci. U. S. A.* **110**, 10095−10100 (2013).
